# Effects of Geometric and Crystallographic Factors on the Reliability of Al/Si Vertically Cracked Nanofilm/Substrate Systems

**DOI:** 10.3390/ma14133570

**Published:** 2021-06-25

**Authors:** Jee S. Shim, Dong H. Go, Hyeon G. Beom

**Affiliations:** Department of Mechanical Engineering, Inha University, 100 Inha-ro, Incheon 22212, Korea; dogfin8@inha.edu (J.S.S.); 22211422@inha.edu (D.H.G.)

**Keywords:** Al/Si bi-material, crystallographic orientation, vertical crack, mechanical properties, fracture mechanism, atomistic simulation

## Abstract

In this study, tensile tests on aluminum/silicon vertically cracked nanofilm/substrate systems were performed using atomistic simulations. Various crystallographic orientations and thicknesses of the aluminum nanofilms were considered to analyze the effects of these factors on the reliability of the nanofilm/substrate systems. The results show that systems with some specific crystallographic orientations have lower reliability compared to the other orientations because of the penetration of the vertical crack into the silicon substrate. This penetration phenomenon occurring in a specific model is related to a high coincidence of atomic matching between the interfaces in the model. This high coincidence leads to a tendency of the interface to maintain a coherent form in which the outermost silicon atoms of the substrate that are bonded to the aluminum nanofilm tend to stick with the aluminum atoms under tensile loads. This phenomenon was verified by interface energy calculations in the simulation models.

## 1. Introduction

Thin-film/substrate systems have been utilized as the fundamental structure for electronic packaging materials and micro/nano-electromechanical systems (M/NEMS). The most commonly utilized material as a substrate is silicon (Si) owing to the feasibility of converting Si into a semiconductor [1,2,3]. The fabrication of thin-film/substrate systems is typically performed by depositing materials onto Si substrates. During the fabrication process, vertical cracks can be nucleated in the system owing to external impact or residual stress between the interfaces. If a vertical crack is nucleated in the system, the system becomes susceptible to external conditions and can easily undergo failure. Therefore, numerous analyses of vertically cracked thin-films bonded to substrate systems have been conducted. Lee et al. [4] developed a mathematical formulation for the penetration of vertical cracks into epitaxial film/substrate systems caused by residual stress between the interfaces. Beom and Jang [5] analyzed a steady-state channeling crack in an orthotropic thin-film bonded to an orthotropic substrate to determine the essential material parameters required to obtain the energy release rate.

The types of thin-film material deposited on Si substrates include polycrystalline Si, Si dioxides, Si nitrides, and aluminum (Al) [2]. Among these materials, Al-Si bi-materials have received much attention because of their exceptional material properties, in particular their low friction coefficient and wear rate [6,7], excellent strength-to-weight ratio and mechanical properties [8,9], low coefficient of thermal expansion, and excellent thermal conductivity [10,11]. Owing to these outstanding and useful properties, various types of research have been performed on the utilization of Al-Si bi-materials for MEMS and sensor structures. Xie et al. [12] fabricated a temperature-insensitive MEMS pressure sensor by utilizing an Al-Si hybrid structure. Zhu et al. [13] proposed a multifunctional sensor utilizing an innovative Al-coated Si wafer bonding method based on polypropylene carbonate and graphite.

As the designs of thin-film/substrate systems are refined down to the nanoscale with the advancement of technology, it becomes necessary to understand the mechanical properties and behaviors of nanomaterials, which differ from those of the materials at the macro- or micro-scale [14]. However, thin-film/substrate systems have yet to be analyzed at the atomic scale owing to the difficulty in directly observing the atomic behaviors of materials. To overcome this difficulty, atomistic simulations could be a suitable solution. Ward et al. [15] analyzed the interfacial deformation and failure behaviors of Al/Si bi-materials and nanocomposites with various crystallographic orientations using atomistic simulations. Zhuo et al. [16] analyzed the interfacial fractures of Al/Si bi-materials with three types of crystallographic orientations via molecular dynamics simulations and compared the simulated normal traction-separation curves to three types of cohesive zone models. However, these studies were restricted to analyses of interfacial fractures between Al and Si. Despite the risk of vertical cracks, an atomistic analysis with respect to the mechanical properties and behaviors of vertically cracked Al nanofilms bonded to Si substrate systems has yet to be performed. Furthermore, the effects of geometric and crystallographic factors on the reliability of Al/Si vertically cracked nanofilm/substrate systems have yet to be analyzed.

Hence, in this work, tensile tests of Al/Si vertically cracked nanofilm/substrate systems were conducted via atomistic simulations. Various crystallographic orientations and thicknesses of the Al nanofilms were considered to analyze their effects on the reliability and mechanical behaviors of the nanofilm/substrate systems. The tensile tests on the nanofilm/substrate systems were conducted in the vertical direction of the crack surface. The mechanical properties and behaviors of the Al/Si vertically cracked nanofilm/substrate systems were obtained and analyzed. The results from a specific simulation model showed some singularities that are absent from the other models.

## 2. Details of Atomistic Simulations

The tension tests of the Al/Si vertically cracked nanofilm/substrate systems were conducted using an atomistic simulation program called the LAMMPS [17,18,19]. In atomic computational simulations, the potential energy of the atoms constituting the simulation models is crucial because the potential energy affects the interactions of the atomic bonds in the simulation models. Ab initio simulations are suitable for obtaining reliable results of atomistic simulations [20,21]. To perform ab initio simulations, however, high-performance computers are required. To reduce the computational burden, the use of empirical potentials is an appropriate alternative to ab initio simulations. In this work, the potential energy for three types of interatomic bonds, namely, Si–Si, Si–Al, and Al–Al bonds, were defined. To reproduce the atomic behaviors involving the three types of interatomic bonds reliably, the MEAM potential was utilized as the empirical potential in this work [22,23]. The MEAM has been verified to be a reliable empirical potential for reproducing the mechanical behaviors in Al-Si bi-material models in earlier studies [15,16,24,25]. In addition, mechanical properties calculated from MEAM potential are known to match well with experimental values [26,27], as presented in Table 1.

The original lattice parameters of Si and Al are known to be 0.5431 nm and 0.405 nm, respectively. A discrepancy, therefore, exists between the two lattice parameters. This results in lattice mismatch between the interfaces when the Al and Si atoms bond to one another. To alleviate the misfit stress caused by the lattice mismatch at the interfaces, the Si lattice parameter was modified to 0.54 nm in previous studies [16,24,25]. This modification was also applied in this work. To investigate the effects of the crystallographic orientations on the mechanical behaviors of the materials, four models, namely, the Al(010)/Si(010), Al(110)/Si(010), Al(010)/Si(110), and Al(110)/Si(110) models, were investigated. Here, the numbers in the brackets indicate the crystallographic orientation with respect to the interface plane of each material.

**Figure 1:**
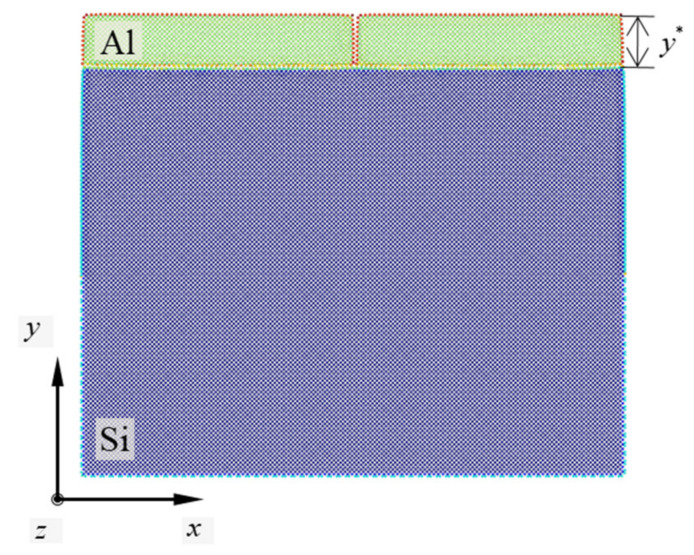
Representative simulation model as the minimum energy states. Si and Al are used as the substrate and nanofilm materials, respectively. The crystallographic orientations with respect to the interface plane of the materials are Al(110) and Si(010). The thickness of the Al nanofilm is approximately 3 nm. The vertical crack is located in the center of the Al nanofilm along the *x*-direction.

All the Cartesian coordinate lengths of the simulation models utilized in this work are presented in Table 2. To investigate the effects of the nanofilm thickness on the mechanical behavior and stability of the nanofilm/substrate systems, seven lengths along the *y*-direction of each Al nanofilm were considered. The variable y* in the table indicates the thickness of the Al nanofilm along the *y*-direction. y* ranges from approximately 2–8 nm in increments of approximately 1 nm. The plane strain conditions were maintained throughout all the simulations by imposing a periodic condition in the *z*-direction of the simulation models. The non-periodic condition was applied in the other Cartesian coordinate directions of the simulation models. To introduce the atomic configuration of the simulation model and the related expressions, a representative simulation model is presented in Figure 1. In this figure, the crystallographic orientations with respect to the interface plane of the materials are Al(110) and Si(010). The exact thickness of the Al nanofilm is 3.15 nm, which will be described as “approximately 3 nm” in the later expressions for the sake of brevity. The color of the atoms in the atomic configurations indicates the potential energy of each material to facilitate the recognition of the vertical crack in the Al nanofilm.

The vertical crack in the simulation models was located at the center of the Al nanofilm along the *x*-direction. The crack was created by screening the atomic interactions between the Al atoms. The length of the pre-crack was set to the thickness of the Al nanofilm in all the simulations. Before performing the tensile tests, all the simulation models were relaxed by minimizing the energy states. The conjugate gradient (CG) method was used as the relaxation method [28]. The tolerance parameter in the stopping criteria for the CG method was set to 10^−24^ eV/nm in this study. In the tensile tests, a single lattice of Si was set as the boundary region at both ends of the simulation models along the *x*-direction. The axial tension along the *x*-direction was performed by applying displacement to each boundary region and then relaxing the simulation model repeatedly. The strain in the tensile tests was maintained at 10^−3^ during the simulations. The temperature of the simulation was set at 0 K. All the simulation procedures were observed via the OVITO software [29].

## 3. Results and Discussion

### 3.1. Stress–Strain Curves

**Figure 2:**
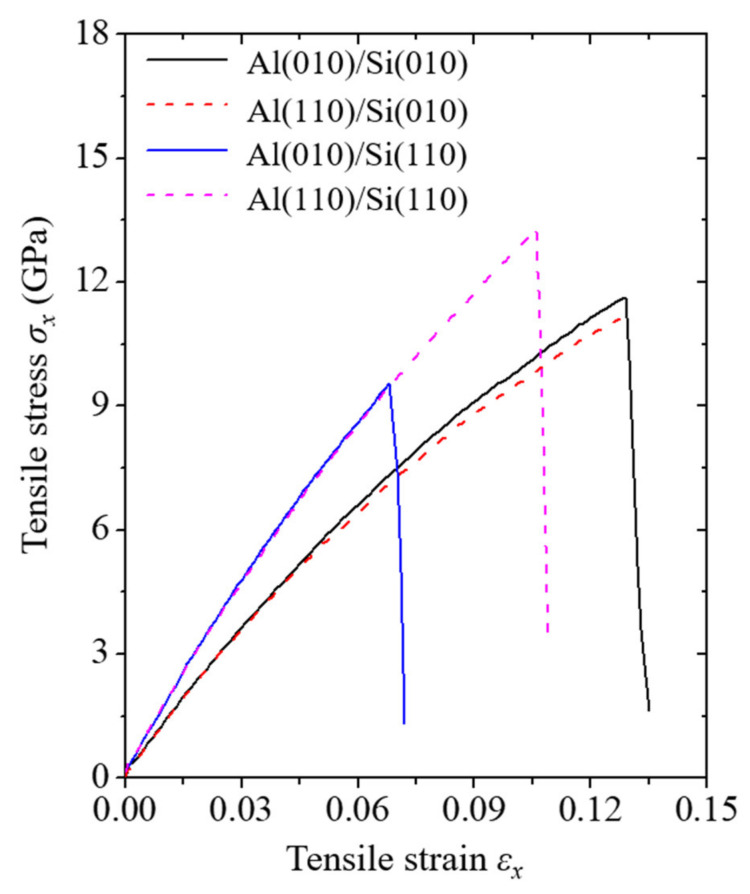
Stress–strain curves of the four simulation models under tension. The nanofilm thickness in the simulation models used for the stress curves is approximately 3 nm.

To analyze the Al/Si vertically cracked nanofilm/substrate systems under tensile stress, the stress curves corresponding to the tensile strain of each simulation model with a nanofilm thickness of approximately 3 nm are plotted in Figure 2. All the stress tensors in the atomistic simulations were calculated using the virial stress formulation [30]. Subramaniyan and Sun [31] have confirmed that the virial stress in atomic-level physics is identical to the Cauchy stress in continuous solid mechanics. The direction of the tensile stress in the vertical axis of the graph in the figure is along the x-direction. In addition, the tensile strain in the horizontal axis of the graph has the same direction as the tensile stress. The engineering strain is utilized as the tensile strain in the figure.

All the stress curves from the simulation models under tension can be divided into two stages: the increasing and falling stages. In the increasing stage, the stress curves for all the simulation models exhibit nonlinear behavior. Specifically, the stress curves exhibit hyperelastic behavior, and this nonlinearity becomes significant as the tensile strain increases. This tendency is a fundamental characteristic of nanostructured Si under tensile load, which has been reported in previous research on Si nanofilms [14] and Si nanowires [32,33]. The tendency of the stress curves indicates that the vertically cracked Al nanofilm does not significantly affect the fundamental characteristics of Si.

Comparing the results of the four simulation models, the stress curves increase with similar trends in the initial slopes between the Si(010) models and between the Si(110) models. The initial slope of a stress curve is equivalent to the Young’s modulus, which is the mechanical property relating the stress and the strain. These similar tendencies of the Young’s moduli for the same substrates may indicate that the Young’s modulus tends to be more sensitive to the crystallographic orientation of the Si substrate than to that of the deposited nanofilm. In this figure, the Young’s moduli of the Al(010)/Si(010) and Al(110)/Si(010) models were calculated to be 116.84 and 122.13 GPa, respectively, and those of the Al(010)/Si(110) and Al(110)/Si(110) models were calculated to be 159.22 and 155.66 GPa, respectively. The Young’s moduli of the nanofilm/substrate systems calculated in this work are similar to those in previous experimental work [34], which analyzed the tension of only the Si film. This result indicates that the vertically cracked nanofilm does not significantly affect the Young’s modulus of the Si substrate when the thickness of the vertically cracked nanofilm is thin. However, it was confirmed that as the vertically cracked nanofilm becomes thicker, the Young’s modulus of the nanofilm/substrate system tends to decrease. In other words, the vertical crack has an increasing influence on the reliability of the nanofilm/substrate system as the length of the vertical crack increases. The Young’s moduli of the Si(010) and Si(110) models across the thicknesses of the Al nanofilms studied in this work were calculated to be about 103.72 to 126.86 GPa, and about 131.98 to 163.15 GPa, respectively.

As the tensile strain exceeds a critical point that signifies the onset of the falling stage, a drastic decrease occurs in the stress curves. In other words, brittle fracture behavior occurs in all the simulation models. The unique point here is that a conspicuous discrepancy is observed at positions of their critical points. The presence or absence of the discrepancy in the critical point position depends on the crystallographic orientation of the Si substrate. Specifically, the critical points of the Si(010) substrate models are located similarly, whereas conspicuous discrepancy is observed at the critical points of the Si(110) substrate models. Detailed discussions of these results will be elucidated in the following sections on the fracture mechanisms and mechanical properties. The stress curve results show that the reliability of the Si(010) models is insensitive to the crystallographic orientation of the deposited nanofilm. In contrast, the reliability of the Si(110) models is sensitive to the crystallographic orientation of the deposited nanofilm. In particular, the Al(010)/Si(110) model shows the most vulnerable reliability among the four simulation models.

### 3.2. Fracture Mechanisms

**Figure 3:**
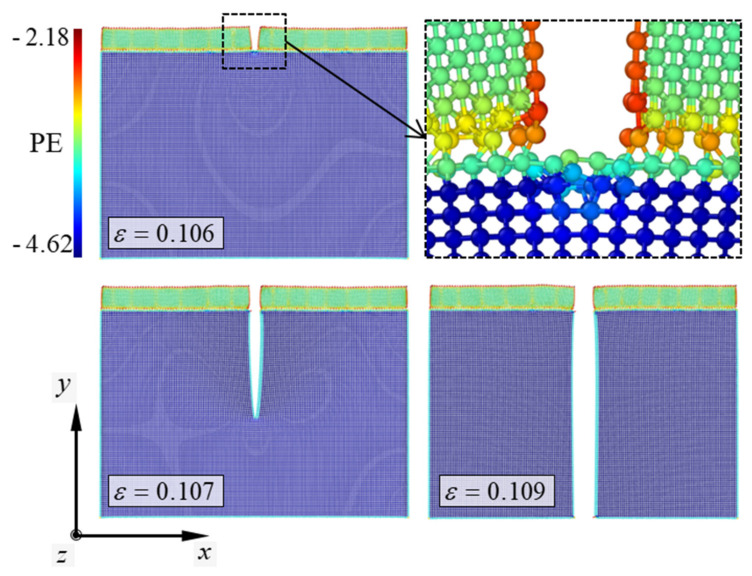
Atomic configurations depicting the fracture mechanism of the Al(110)/Si(110) model. The nanofilm thickness in the simulation model is approximately 3 nm. The PE indicates the atomic potential energy of the materials.

**Figure 4:**
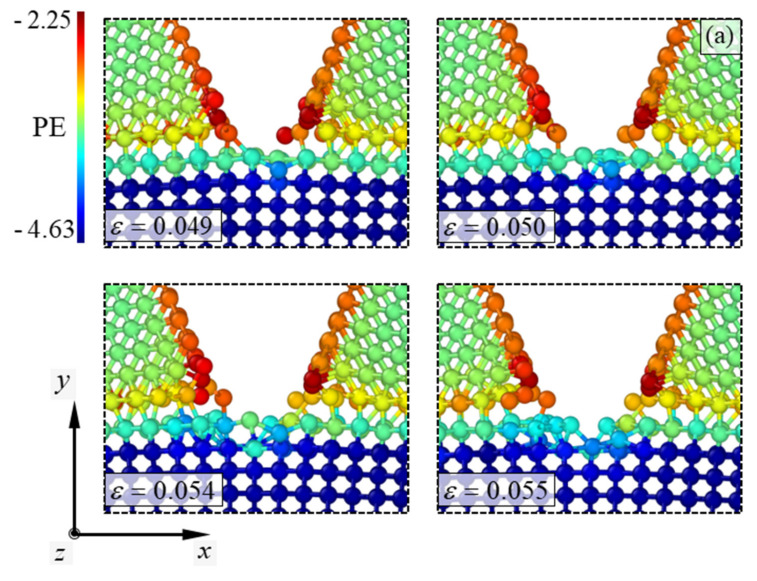

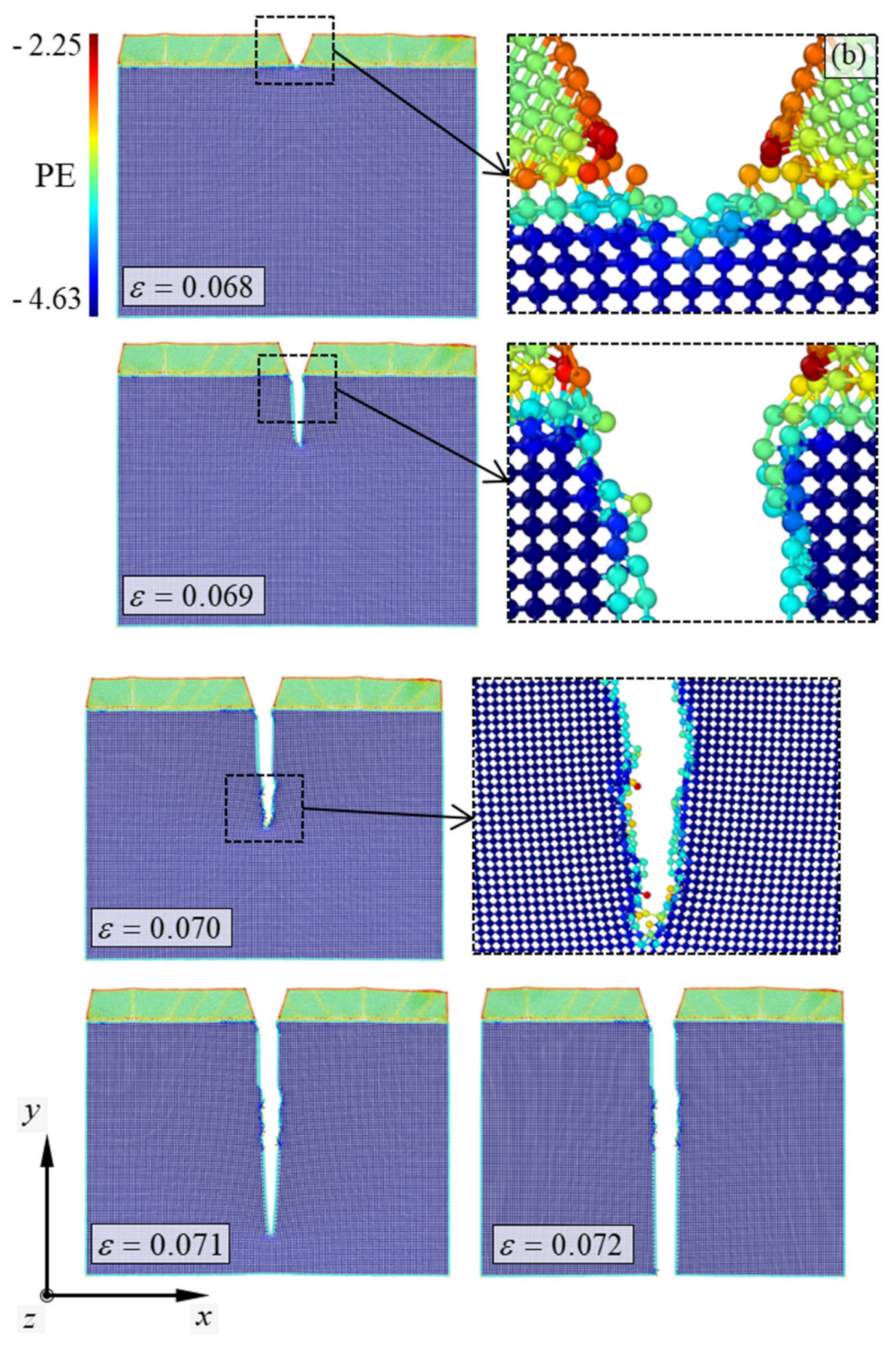
Atomic configurations depicting the fracture mechanism of the Al(010)/Si(110) model. The nanofilm thickness in the simulation model is approximately 3 nm. The PE indicates the atomic potential energy of the materials. (**a**) depicts penetration phenomenon of vertical crack into the Si substrate, and (**b**) depicts crack propagation in the Si substrate.

To study the effects of the crystallographic orientations on the fracture mechanisms of the simulation models, the atomic configurations depicting the mechanical behaviors of the Al/Si vertically cracked nanofilm/substrate systems under tension are presented in Figure 3 and Figure 4. The selected nanofilm thickness of the plotted atomic configurations is approximately 3 nm, similar to the stress curves shown earlier. As previously mentioned, the fracture mechanism of the Al(010)/Si(110) model shows singularities that are absent in the other simulation models. In contrast, the other models, i.e., Al(010)/Si(010), Al(110)/Si(010), and Al(110)/Si(110), commonly show a sudden brittle cleavage in the Si substrates without noticeable deformation when the strain in the simulation models exceeds a critical value. As these three simulation models exhibit similar failure behaviors, only a single fracture mechanism is shown for these models.

The atomic configurations in Figure 3 depict the failure behavior of the Al(110)/Si(110) model near the critical strain. The color of the atoms in the configurations indicates the atomic potential energy in the simulation model, which ranged from approximately −4.62 to −2.18 eV. In the atomic configurations, the vertical crack of the Al nanofilm maintained a square form without noticeable deformation before brittle cleavage occurred in the Si substrate. In other words, the vertical cracks of the Al nanofilm did not penetrate the Si substrate in the failure behavior of the Al(110)/Si(110) model. This result is a normal phenomenon because Si is generally stiffer than Al. When the critical strain of 0.106 was exceeded in the simulation model, brittle cleavage occurred suddenly in the Si substrate. The brittle cleavage was initiated at the center of the crack tip. The crack propagated in the vertical direction of the tensile load. When the strain in the simulation model exceeded 0.109, the Si substrate was broken into two pieces owing to crack propagation, and the failure behavior of the simulation model ended. The brittle cleavage caused by crack propagation results in the formation of a $\left(\bar{1}10\right)$ surface on the Si substrate. The newly formed plane has a flat and clean form without any tear. A fracture surface with the same form was reported in a previous study [14], which showed that the newly formed surfaces in fractured Si nanofilms belonged to the {110} family.

In contrast to the fracture mechanisms of the other simulation models, the Al(010)/Si(110) model showed a singularity before crack propagation occurred in the Si substrate. The atomic configurations depicting the fracture mechanisms of the Al(010)/Si(110) model are presented in Figure 4. Similar to Figure 3, the color of the atoms in the configurations indicates the atomic potential energy of the material, which in the Al(010)/Si(110) model ranged from about −4.63 to −2.25 eV. When the strain in the Al(010)/Si(110) model exceeded 0.049, the vertical crack in the Al nanofilm initiated to penetrate the Si substrate. When the strain exceeded 0.055, the vertical crack penetrated the Si substrate by breaking the atomic bonds between the outermost atoms of the Si substrate. This penetration of the vertical crack caused the nucleation of a tiny edge crack in the Si substrate. The extended crack, which was aligned with the crack surface of the Al nanofilm, maintained its form until fracture occurred in the Si substrate. When the strain exceeded 0.068, the crack in the Si substrate initiated propagation. The initial propagation of the crack was not parallel to the boundary regions of the Si substrate but instead had a slightly kinked form. When the strain exceeded 0.069, the crack soon propagates parallel to the boundary regions of the Si substrate. Similar to the Al(110)/Si(110) model, a $\left(\bar{1}10\right)$ surface was formed on the Si substrate as the crack propagated. However, when the strain exceeded 0.07, the crack propagation of the Al(010)/Si(110) model resulted in the formation of a torn plane instead of a clean and flat one on the newly formed surface. Nonetheless, the crack propagation soon resulted in the formation of a clean plane at the Si substrate when the strain exceeded 0.071. When the strain in the model exceeded 0.072, the propagation of the crack ended, and the Si substrate was broken into two pieces.

**Figure 5:**
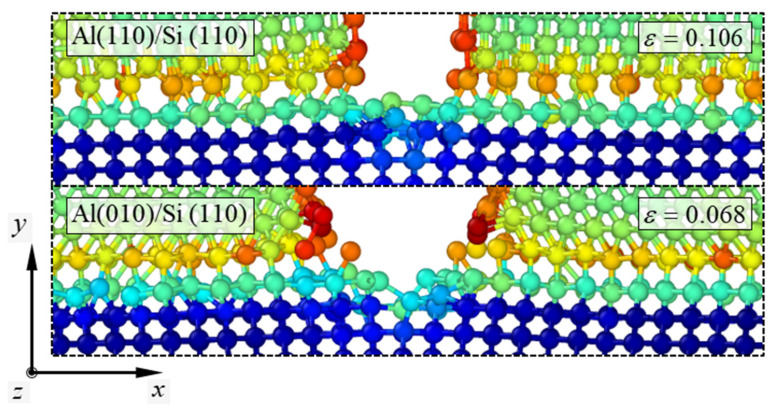
Atomic configurations of the interfaces in the Al(110)/Si(110) and Al(010)/Si(110) models at the failure strain. The color of the atoms indicates the atomic potential energy. The nanofilm thickness is approximately 3 nm.

Comparing the failure strain of the Al(010)/Si(110) model with that of the other models, it can be seen that the edge crack nucleation in the Si substrate is a fatal flaw in the reliability of the nanofilm/substrate system. This reduction in the reliability of the nanofilm/substrate systems has been observed in the stress–strain curves described earlier and will be verified in the following section. The noticeable discrepancy between the Al(010)/Si(110) model and the other models is the coincidence level of atomic matching between the interfaces (Figure 5).

The atomic configurations of the Al(010)/Si(110) model exhibit a coherent interface in which the Al atoms and Si atoms at the interface have a one-on-one bonding pattern. The fundamental reason behind the vulnerability of the Al(010)/Si(110) model compared to the other models is likely the coherence of the interface. According to previous studies [35,36], interface planes with a high coincidence of atom-row matching tend to maintain a coherent form. This results in a tendency for the outermost Si atoms bonded to the Al nanofilm to move along with the Al atoms when the system is under tensile load. Therefore, a tiny edge crack can be nucleated even below the critical strain by breaking the bonds between the outermost Si atoms at the crack tip. This phenomenon was verified by comparing the interface energies of the simulation models, as described below. According to previous studies [36,37], an interface with a high coincidence of atom-row matching has minimal interface energy because of the tendency to form a coherent interface. To verify this trend, the interface energies of the Al(010)/Si(110) and Al(110)/Si(110) models were calculated. The calculation of the interface energy has been studied in previous work [38]. The expression for the interface energy $\gamma_{\mathrm{int}}$ used in this work is:

(1) $$\gamma_{\mathrm{int}}=\frac{U-(N^{\mathrm{Al}}U_{coh}^{\mathrm{Al}}+N^{\mathrm{Si}}U_{coh}^{\mathrm{Si}})}{2A},$$

where *U* is the total energy of the nanofilm/substrate system in a minimized state; $N^{\mathrm{Al}}$ and $N^{\mathrm{Si}}$ indicate the number of Al and Si atoms, respectively; $U_{coh}^{\mathrm{Al}}$ and $U_{coh}^{\mathrm{Si}}$ indicate the cohesive energy with respect to the atomic bonds of Al and Si atoms, respectively; and A indicates the area of the interface. The coefficient of A indicates that there are two planes between the interfaces. The cohesive energy for the calculation was obtained from the values of a previous study [23]. The interface energies of the Al(010)/Si(110) and Al(110)/Si(110) models in this work were calculated to be approximately 3.379 and 3.57 J/m^2^, respectively. The interface energy of the Al(010)/Si(110) model has a relatively low value, whereas the Al(110)/Si(110) model shows the highest interface energy among all the simulation models. These results support the hypothesis that the outermost Si atoms bonded to the Al nanofilm of the Al(010)/Si(110) model have a tendency to maintain bonds with the Al atoms, and the interface plane of the Al(010)/Si(110) model tends to act as a single component. Therefore, tiny edge cracks can be nucleated in the Si substrate of the Al(010)/Si(110) model, even below the critical strain, through the breaking of the atomic bonds between the outermost Si atoms at the crack tip.

### 3.3. Mechanical Properties

**Figure 6:**
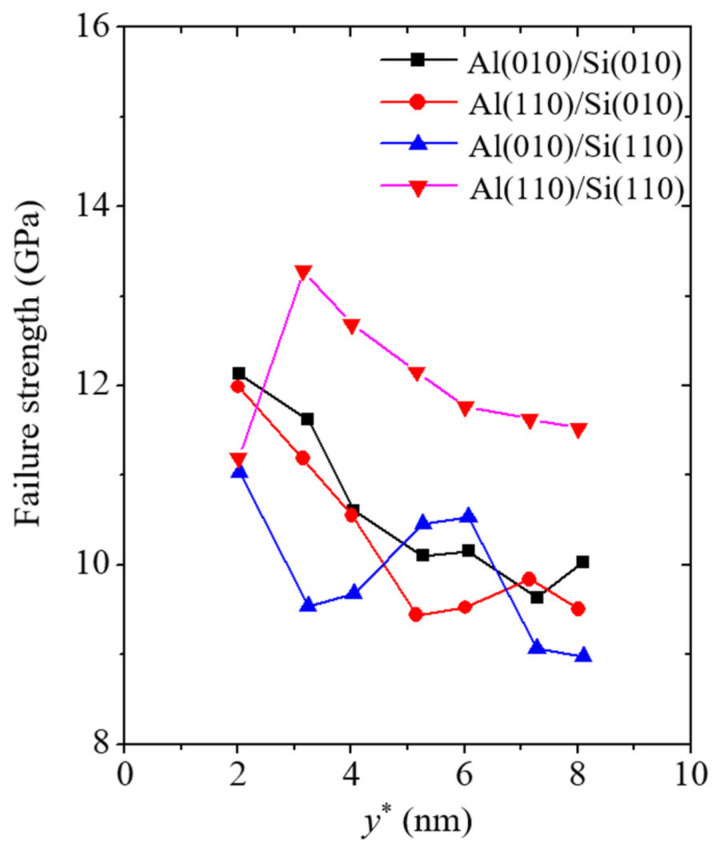
Failure strength corresponding to the varying thicknesses of Al nanofilm denoted by the variable y*.

**Figure 7:**
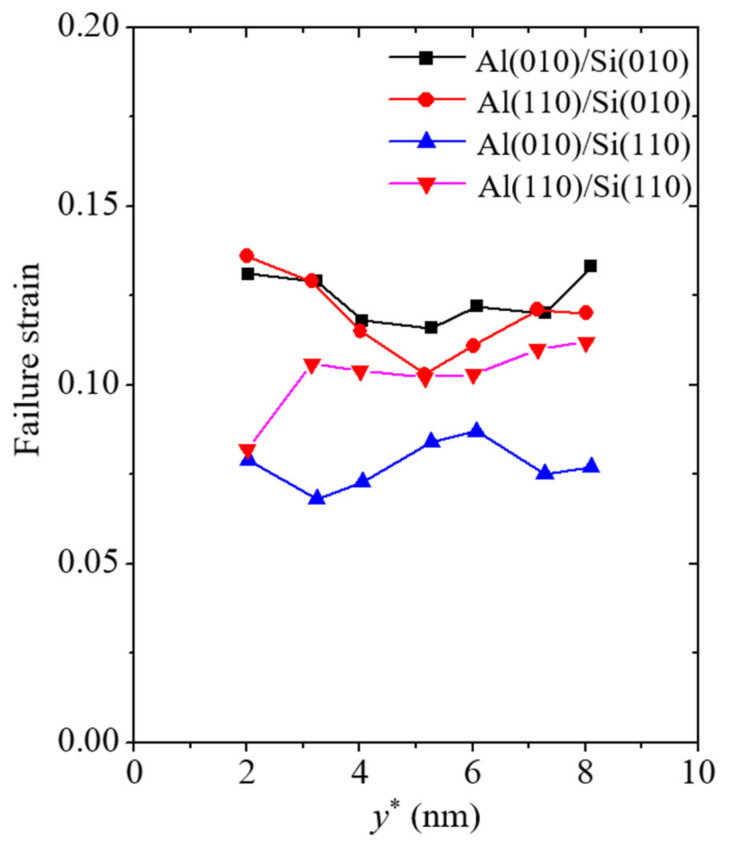
Failure strain corresponding to the varying thicknesses of Al nanofilm denoted by the variable y*.

In addition to the Young’s modulus described earlier, the failure strength, failure strain, and fracture energy of the simulation models were calculated to analyze the reliability of the vertically cracked nanofilm/substrate systems. The failure strength and strain are basic and important mechanical property indices for the reliability of materials. The failure strength and strain were taken as the values of the strength and strain when the failure of the material initiated, respectively. To analyze the effects of geometric factors on the mechanical properties of the nanofilm/substrate systems, the thickness of the Al nanofilm was varied in this section. The failure strength and strain corresponding to the varying values of the Al nanofilm thickness y* are plotted in Figure 6 and Figure 7, respectively.

The failure strengths of all the simulation models show size-dependent characteristics (Figure 6). The failure strength tends to decrease as the thickness of the Al nanofilm increases. This tendency indicates that the vertical crack in the Al nanofilm becomes a fatal flaw for the reliability of the system as the length of the vertical crack increases. The calculated failure strengths of the Al(010)/Si(010) and Al(110)/Si(010) models range from 9.6 to 12.2 GPa and 9.4 to 12 GPa, respectively. No noticeable discrepancy between the two Si(010) models was observed. The failure strengths from the simulation models are similar to those of previous experimental work [39] in which tensile tests on Si nanowires with a diameter of approximately 100 nm were performed. These results indicate that the Si(010) substrate tends to be insensitive to the crystallographic orientation of the deposited nanofilm, as mentioned previously. However, a conspicuous discrepancy was observed upon comparing the two Si(110) models. In the two Si(110) models, the failure strength ranges of the Al(010)/Si(110) and Al(110)/Si(110) models were calculated to be 8.9 to 11.1 GPa and 11.1 to 13.3 GPa, respectively. Except at the nanofilm thickness y* of approximately 2 nm, the Al(110)/Si(110) model shows higher durability than the Al(010)/Si(110) model. The conspicuous discrepancy between the two Si(110) models here is related to the penetration of the vertical crack. The previous results have shown that the nucleation of tiny edge cracks in the Si substrate is a fatal flaw in the Al(010)/Si(110) model. However, when the thickness of the Al nanofilm is less than 3 nm, the vertical crack in the nanofilm does not significantly affect the nanofilm/substrate system because of the size of the nanoscale unit.

A similar trend was observed in the failure strain results of all the simulation models (Figure 7). The failure strain ranges of the Al(010)/Si(010) and Al(110)/Si(010) models are 0.116 to 0.133 and 0.103 to 0.136, respectively, while those of the Al(010)/Si(110) and Al(110)/Si(110) models are 0.068 to 0.087 and 0.082 to 0.112, respectively. The Al(010)/Si(110) model has the lowest failure strain among all the models because of the penetration of the vertical crack into the Si substrate in this model before the occurrence of fracture. In contrast, the failure strains of the other simulation models have similar values to those found in previous experimental work [39]. This means that the failure strain of the nanofilm/substrate systems is insensitive to the vertical crack in the nanofilm when the vertical crack does not penetrate the substrate. In addition, no noticeable size-dependent failure strain characteristics were observed in the simulation models.

**Figure 8:**
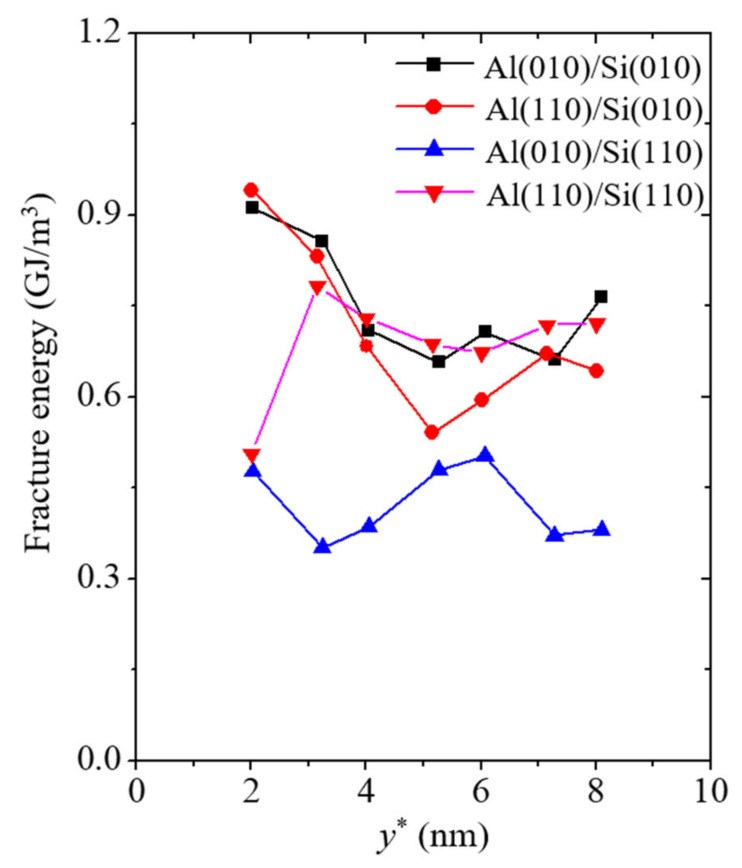
Fracture energy corresponding to the varying thicknesses of Al nanofilm denoted by the variable y*.

The last mechanical property to be elucidated is the fracture energy. It is related to the amount of energy that material under tensile loads can absorb. It can be obtained by integrating the tensile stress up to the critical strain [40]. The fracture energy G* can be expressed as

(2) $$G^{*}=\int_{0}^{\varepsilon_{x}^{c}}\sigma_{x}d\varepsilon_{x},$$

where $\varepsilon_{x}^{c}$ and $\sigma_{x}$ are the critical strain and the tensile stress along the tension direction, respectively. The SI unit of the fracture energy is GJ/m^3^. The fracture energy corresponding to the various thicknesses of the Al nanofilm is plotted in Figure 8. The ranges of the fracture energy in the Al(010)/Si(010) and Al(110)/Si(010) models are 0.657 to 0.911 GJ/m^3^ and 0.54 to 0.941 GJ/m^3^, respectively, while those in the Al(010)/Si(110) and Al(110)/Si(110) models are 0.352 to 0.503 GJ/m^3^ and 0.506 to 0.783 GJ/m^3^, respectively. Similar tendencies with the failure strength and strain were observed for the fracture energy. Compared to the fracture energy of the other simulation models, the Al(010)/Si(110) model has the lowest fracture energy because of the penetration phenomenon of the vertical crack before the occurrence of fracture behavior. The absence of atomic bonds between the outermost Si atoms in the Al(010)/Si(110) model reduces the ability of the system to bear external loads. Additionally, a slight size dependence was observed in the fracture energy results of the simulation models. As the thickness of the Al nanofilm increases, the fracture energies of the simulation models tend to decrease slightly. This size dependence is due to the inclusion of the failure strength, which is size-dependent, in the calculation of the fracture energy.

## 4. Conclusions

To analyze the effects of geometric and crystallographic factors on the reliability of vertically cracked nanofilm/substrate systems, tensile tests were conducted on Al/Si vertically cracked nanofilm/substrate systems using atomistic simulations. The MEAM potential was employed to reproduce the interactions of the atomic bonds in the simulation models. Four simulation models with different crystallographic orientations were studied. In addition, Al nanofilms of various thicknesses were modeled, and their effects on the mechanical properties and behaviors of the Al/Si vertically cracked nanofilm/substrate systems were analyzed. The results of the simulations show that Young’s modulus of the Al/Si vertically cracked nanofilm/substrate system tends to be more sensitive to the crystallographic orientation of the Si substrate than to that of the deposited nanofilm. The failure strength, strain, and fracture energy results show some singularities. Specifically, the Al(010)/Si(110) model has the lowest reliability because of the penetration of the vertical crack into the Si substrate before fracture behavior occurs. This penetration phenomenon in the Al(010)/Si(110) model is related to the high coincidence of atom-row matching between the interfaces in the model. This high coincidence results in a tendency to maintain the coherent form to minimize the interfacial energy with which the outermost Si atoms that are bonded to the Al nanofilm tend to stick to the Al atoms under tensile loads. Consequently, a tiny edge crack can be nucleated in the Si substrate of the Al(010)/Si(110) model even below the critical strain, while the absence of atomic bonds between the outermost Si atoms at the crack tip of the model makes the system unable to bear external loads. This phenomenon was verified by the interface energy calculations of the simulation models.

## Figures and Tables

**Table 1:** Elastic constants of Al and Si. The SI unit of the elastic constant *C_ij_* is GPa.

	Al		Si	
	MEAM	Experiment [26]	MEAM	Experiment [27]
*C*_11_	110.81	116.3	165.63	165.64
*C*_12_	61.1	64.8	63.88	63.94
*C*_44_	28.72	30.9	83.93	79.51

**Table 2:** Lengths of the simulation models in Cartesian coordinates. The variable y* denotes the length of the Al nanofilm along the *y*-direction. The numbers in the brackets denote the crystallographic orientation with respect to the interface plane of each material.

Length (nm)	Si(010)		Si(110)		Al(010)	Al(110)	
*x*	32.4		32.456		32.4	32.361	
*y*	24.3		24.438		y*	y*	
*z*	1.62		1.62		1.62	1.62	
y* of Al(010)	2.025	3.24	4.05	5.265	6.075	7.29	8.1
y* of Al(110)	2.005	3.15	4.009	5.155	6.014	7.159	8.019

## Data Availability

Data sharing is not applicable for this article.
